# Supplementary material for: Exploring U.S. Food System Workers’ Intentions to Work While Ill during the Early COVID-19 Pandemic: A National Survey
Source: Int J Environ Res Public Health. 2023 Jan 16;20(2):1638. doi: 10.3390/ijerph20021638 (PMC9865134; doi:10.3390/ijerph20021638)
Supplement: Supplementary file 1 [file ijerph-20-01638-s001.zip › Table S3.pdf]

Table S3. Sensitivity analyses for associations between variables of interest and odds ratio for COVID-19 presenteeism intentions within a national sample of food system workers in the U.S.

Subgroup:	Respondents reporting customer interaction	Respondents who were "Required" to work	Accounting for state-level clustering
	Odds Ratio<br>95% CI<br>P value<br>N=1171	Odds Ratio<br>95% CI<br>P value<br>N=664	Odds Ratio<br>95% CI<br>P value
**Organizational Safety Climate Score**			
Low	Ref	Ref	Ref
High	0.70<br>0.43, 1.11<br>0.13	0.53<br>0.31, 0.90<br>0.02	0.63<br>0.42, 0.94<br>0.02
**Quantitative Work Demands**			
Low	Ref	Ref	Ref
High	1.52<br>0.93, 2.51<br>0.10	1.29<br>1.73, 2.27<br>0.38	1.51<br>1.00, 2.30<br>0.05
**USDA Food Security Category**			
High	Ref	Ref	Ref
Low	1.52<br>0.85, 2.76<br>0.16	1.26<br>0.66, 2.41<br>0.48	2.18<br>1.35, 3.50<br>0.001
Very low	2.87<br>1.62, 5.08<br><0.001	1.39<br>0.72, 2.71<br>0.33	2.37<br>1.41, 3.98<br>0.001
**Access to paid leave**			
No	Ref	Ref	Ref
Yes	1.22	0.82	1.18

	0.71, 2.08	0.44, 1.53	0.74, 1.87
	0.71	0.53	0.49
**"It is worth the health risk to reopen the economy as soon as possible"**			
Strongly/disagree	Ref	Ref	Ref
Neutral	1.45	1.99	1.47
	0.85, 2.49	1.07, 3.69	0.93, 2.32
	0.18	0.03	0.10
Strongly/Agree	2.57	2.63	2.40
	1.46, 4.52	1.37, 5.05	1.47, 3.92
	0.001	0.004	<0.001
**Food System Sector**			
Retail	Ref	Ref	Ref
Production	3.66	2.50	3.73
	1.03, 13.04	0.69, 9.13	1.72, 8.11
	0.05	0.16	0.001
Processing	0.73	0.80	1.19
	0.15, 3.56	0.31, 2.08	0.54, 2.62
	0.70	0.65	0.67
Distribution	2.10	1.97	2.31
	0.66, 6.68	0.56, 6.94	0.85, 6.26
	0.21	0.29	0.10
Restaurant/Service	1.03	0.86	1.25
	0.56, 1.90	.40, 1.86	0.72, 2.17
	0.92	0.70	0.43
Food Assistance	0.51	0.40	0.43
	0.12, 2.41	.07, 2.21	0.09, 1.99
	0.40	0.30	0.28
