# Supplementary material for: Exploring U.S. Food System Workers’ Intentions to Work While Ill during the Early COVID-19 Pandemic: A National Survey
Source: Int J Environ Res Public Health. 2023 Jan 16;20(2):1638. doi: 10.3390/ijerph20021638 (PMC9865134; doi:10.3390/ijerph20021638)
Supplement: Supplementary file 1 [file ijerph-20-01638-s001.zip › Table S4.pdf]

Table S4. Comparisons between groups reporting presenteeism intentions versus not and variables of interest in a national sample of food system workers during early COVID-19.

		"If I was sick with COVID-19, but I was still able to work, I would go to work."		
		Did not agree	Agree	
		N=2313	N= 222	
		n (%) or Mean (SD)	n (%) or Mean (SD)	p value
**Demographics**				
Age, m(sd)		46.06 (11.27)	43.75 (10.78)	0.004
Race (n=2527)	White	1,991 (86.3)	182 (82.3)	
	African American	100 (4.3)	12 (5.4)	
	Other / Mixed race	215 (9.3)	27 (12.2)	0.26
Ethnicity (n=2440)	Not Hispanic/Latinx	2008 (90.3)	188 (87.4)	
	Hispanic/Latinx	217 (9.8)	27 (12.6)	0.19
Gender (n=2535)	Female	1,521 (65.8)	120 (54.1)	
	Male	754 (32.6)	92 (41.4)	
	Other Answer	38 (1.6)	10 (4.5)	<0.001
Sector (n=2535	Production	87 (3.8)	28 (12.6)	
	Processing	203 (8.8)	24 (10.8)	
	Distribution	52 (2.3)	8 (3.6)	
	Retail	818 (35.4)	66 (29.7)	
	Restaurant/Service	1006 (43.5)	91 (41.0)	
	Food Assistance	147 (6.4)	5 (2.3)	<0.001
Income (n=2330)	< $25,000	586 (27.5)	56 (27.9)	
	$25,000 – 34,999	385 (18.1)	42 (20.9)	
	$35,000 – 49,999	382 (17.9)	45 (22.4)	
	$50,000 – 99,000	45 (30.3)	51 (25.4)	
	> $100,000	131 (6.2)	7 (3.5)	0.17
U.S. Census Region (n=2375)	Northeast	388 (17.9)	39 (19.1)	
	Midwest	599 (27.6)	55 (27.0)	
	South	785 (36.2)	72 (35.3)	
	West	399 (18.4)	38 (18.6)	0.97
**Occupational Attributes**				
Union Status (n=2471)	Non-Union Member	1796 (79.6)	169 (79.0)	
	Union Member	461 (20.4)	45 (21.0)	0.84

Employer Size (n=2454)	1 – 10	288 (12.9)	28 (13.0)	
	11 - 49	745 (33.3)	68 (31.6)	
	50 - 499	1030 (46.0)	90 (41.9)	
	More than 500	176 (7.9)	29 (13.5)	0.04
Full-time/part-time status(n=2332)	Full Time	1376 (64.5)	134 (67.3)	
	Part Time	609 (28.6)	42 (21.1)	
	Other	148 (6.9)	23 (11.6)	0.01
Customer Contact (n=2523)	No	546 (23.7)	59 (26.9)	
	Yes	1758 (76.3)	160 (73.1)	0.29
Access to Paid Leave (n=2527)	No	1660 (72.0)	168 (75.7)	
	Yes	645 (28.0)	54 (24.3)	0.25
Employer granted "easier" access to sick leave (n=2527)	No	1596 (69.2)	170 (76.6)	
	Yes	709 (30.8)	52 (23.4)	0.02
Work Demands (n=2464)		46.0 (24.4)	54.0 (25.7)	<0.001
Safety Climate (n=2373)		17.7 (4.4)	15.9 (4.9)	<0.001
Social Support (n=2287)		67.2 (21.8)	59.4 (23.3)	<0.001
Required to Work (n=2420)	Required	702 (31.2)	90 (42.3)	
	Asked	580 (26.3)	43 (20.2)	
	Both	292 (13.2)	32 (15.0)	
	Neither	633 (28.7)	48 (22.5)	0.006
**Non-occupational attributes**				
Food Security Status since pandemic declaration (n=2374)	High or marginal	1317 (60.7)	82 (40.2)	
	Low	441 (20.3)	64 (31.4)	
	Very low	412 (19.0)	58 (28.4)	<0.001
It is worth the health risk to reopen the economy as soon as possible. (n=2114)	Strongly/Disagree	829 (43.0)	58 (31.4)	
	Neither Agree/Disagree	697 (36.1)	63 (34.1)	
	Strongly/Agree	403 (20.9)	64 (34.6)	<0.001
