# Supplementary material for: Exploring U.S. Food System Workers’ Intentions to Work While Ill during the Early COVID-19 Pandemic: A National Survey
Source: Int J Environ Res Public Health. 2023 Jan 16;20(2):1638. doi: 10.3390/ijerph20021638 (PMC9865134; doi:10.3390/ijerph20021638)
Supplement: Supplementary file 1 [file ijerph-20-01638-s001.zip › Table S6.pdf]

Table S6. Comparison of participants who self-selected to provide additional comments on their decision to attend work with non-commentors and entire sample.

		Commentors	Non-commentors		Total Sample
		n (%) or mean, SD	n (%) or mean, SD	p value	n (%) or mean, SD
Total N		N=460	N=2,939		N=3,399
Sector					
(n=460)	Production	15 (3.3)	155 (5.3)		170 (5.0)
	Processing	39 (8.5)	262 (8.9)		301 (8.9)
	Distribution	21 (4.6)	61 (2.1)		82 (2.4)
	Retail	155 (33.7)	1000 (34.0)		1,155 (33.4)
	Restaurant/ Service	200 (43.5)	1285 (43.7)		1,485 (43.7)
	Assistance	30 (6.5)	176 (6.0)	0.02	206 (6.1)
Age					
(n=460)	Mean, SD	44.5 (11.2)	46.1 (11.3)	0.003	46.0 (11.3)
Gender					
(n=460)	Female	317 (68.9)	1890 (64.3)		2,207 (65.0)
	Male	134 (29.1)	985 (33.5)		1,119 (32.9)
	Other	9 (2.0)	64 (2.2)	0.156	73 (2.2)
Race					
(n=459)	White	386 (84.1)	2504 (85.6)		2,890 (85.4)
	Non-White	73 (15.9)	426 (14.6)	0.525	495 (14.6)
Ethnicity					
(n=448)	Hispanic	43 (9.6)	2514 (89.0)		353 (10.8)
	Non-Hispanic	405 (90.4)	310 (11.0)	0.382	2,919 (89.2)
Income					
(n=445)	<10,000	10 (2.2)	93 (4.8)		103 (4.3)
	10,000 - 15,000	27 (6.1)	150 (7.7)		177 (7.4)
	15,-000 - 24,999	71 (16.0)	310 (15.9)		381 (15.9)
	25,000 - 34,999	88 (19.8)	353 (18.1)		441 (18.5)
	35,000 - 49,999	78 (17.5)	356 (18.3)		434 (18.2)
	50,000 - 74,999	97 (21.8)	385 (19.8)		482 (20.2)
	75,000 - 99,999	56 (12.6)	173 (8.9)		229 (9.6)
	>= 100,000	18 (4.0)	125 (6.4)	0.02	143 (6.0)
U.S. Region					
(n=443)	Northeast	80 (18.1)	488 (17.9)		568 (17.9)
	South	119 (26.9)	758 (27.8)		877 (27.7)
	Midwest	170 (38.4)	975 (35.7)		1145 (36.1)
	West	74 (16.7)	507 (18.6)	0.661	581 (18.3)
Union Membership					
(n=451)	Non-union	354 (78.5)	2285 (80.1)		2639 (79.6)
	Union	97 (21.5)	567 (19.9)	0.423	664 (20.5)
Job Pay Status					

(n=441)	Hourly	325 (73.7)	1468 (75.4)		1793 (75.1)
	Hourly with tips	49 (11.1)	49 (2.5)		195 (8.2)
	Salary	67 (15.2)	333 (17.1)	<0.001	400 (16.8)
Full/Part-time status					
(n=443)	Full time	258 (58.2)	1291 (66.3)		1,549 (64.8)
	Part time	125 (28.2)	544 (27.9)		669 (28.0)
	Other	60 (13.5)	113 (5.8)	<0.001	173 (7.2)
Customer Status					
(n=459)	No customers	105 (22.9)	697 (23.9)		802 (23.8)
	Yes customers	354 (77.1)	2219 (76.1)	0.631	2573 (76.2)
Organization Size					
(n=451)	1 to 10	49 (10.9)	391 (13.8)		440 (13.4)
	11 to 49	157 (34.8)	357 (12.6)		1114 (34.0)
	50 - 499	211 (46.8)	1237 (43.7)		1448 (44.2)
	>=500	34 (7.5)	244 (8.6)	0.26	278 (8.5)
USDA Food Security Score					
(n=414)	High or marginal	234 (56.5)	1065 (60.2)		1299 (59.5)
	Low food security	84 (20.3)	379 (21.4)		463 (21.2)
	Very low food security	96 (23.2)	325 (18.4)	0.082	421 (19.3)
" If I was sick with COVID-19, but I was still able to work, I would go to work"					
(n=2,535)	Agree	40 (9.0)	182 (8.7)		222 (8.8)
	Do not agree	405 (91.0)	1,908 (91.3)	0.849	2,313 (91.2)
